# Decrease in Psoas Muscle Mass and Density Following Liver Transplantation Is Greatest in Patients With the Highest Muscle Quantity and Density Pre‐Transplant

**DOI:** 10.1111/ctr.70410

**Published:** 2025-12-15

**Authors:** Michael G. Megaly, William C. Miller, Jessica Thul, Peter Gullickson, Abraham J. Matar, Michael Dryden, Matthew Wright, David Mathews, Jessica Fisher, Heidi Sarumi, Levi Teigen, Scott Lunos, Timothy L. Pruett

**Affiliations:** ^1^ Division of Solid Organ Transplantation Department of Surgery University of Minnesota Minneapolis Minnesota USA; ^2^ University of Minnesota Medical School Minneapolis Minnesota USA; ^3^ MHealth Fairview University of Minnesota Medical Center Minneapolis Minnesota USA; ^4^ Department of Food Science and Nutrition University of Minnesota St. Paul Minnesota USA; ^5^ Division of Gastroenterology, Hepatology, and Nutrition Department of Medicine University of Minnesota Minneapolis Minnesota USA; ^6^ Biostatistical Design and Analysis Center Clinical and Translational Science Institute University of Minnesota Minneapolis Minnesota USA

**Keywords:** liver transplantation, nutrition, psoas muscle area index: mean Hounsfield units, sarcopenia

## Abstract

Sarcopenia is a known predictor of morbidity and mortality after liver transplantation (LT); it has been assessed with computed tomography (CT) derived Psoas Area Index (PAI) and mean Hounsfield units (mHU). While literature is abundant regarding the adverse outcomes of liver transplant in sarcopenic patients, a paucity of data exists describing the change in psoas muscle area and density from pre‐ to post‐liver transplant. One hundred and four adult liver transplant recipients had pre‐ and post‐transplant CT scans analyzed with respect to PAI and mHU. Mean PAI pre‐transplant was 7.94 and 6.99 cm^2^/m^2^ post‐transplant (12% loss). Mean mHU pre‐transplant was 35.47 and 33.00 post‐transplant (7% reduction). However, stratified by pre‐transplant quartiles, PAI reduction was −15%, −12%, and −6% for the upper, mid‐two, and lower quartiles, respectively (*p* value = 0.0028). The mHU stratification was −15%, −8%, and  + 12% for the upper, mid‐two, and lower quartiles, respectively (*p* value = 0.0004). No relationship was noted between PAI and mHU. PAI and mHU decreased following liver transplantation; however, the most pronounced decrease in muscle mass and density was in patients with the highest starting muscle mass and density. However, muscle mass (PAI) and composition (mHU) appear to be affected by multiple factors.

## 1 Introduction

The number of liver transplants (LT) globally continues to increase on an annual basis, for increasingly diverse indications [1, 2]. Irrespective of indication, diminished muscle mass, sarcopenia, is associated with an increased risk for adverse outcomes [3, 4]. While sarcopenia was initially used to describe the loss of muscle mass associated with aging or immobility, chronic disease, perioperative stresses, and immobility will also diminish muscle mass. Sarcopenia, measured either pre‐ or post‐transplant, has been correlated with poor outcomes [5, 6, 7]. However, there is little information quantifying the loss of muscle occurring during the LT event that would alter the sarcopenia assessment.

Computed tomography (CT) image analysis is a common technique used to assess muscle mass and density [3]. CT‐derived Psoas Area Index (cross‐sectional psoas area at L3/patient height in meters [2], PAI) and mean Hounsfield units (mHU) within the psoas area are data points that measure core skeletal muscle quantity and density, respectively [8, 9, 10, 11, 12]. Since density is an aggregate of protein/myofibrils and those components with a lower mHU, such as fat and water, density is often viewed as a surrogate for muscle quality [8, 9, 10]. The use of intravenous contrast changes the Hounsfield unit density but can be reliably adjusted [13]. Additionally, computerized analysis of abdominal CT scans has expanded the information provided about body composition. However, when focusing on abdominal muscle mass, it is very comparable to localized PAI at L3 [14].

This study is designed to assess changes in muscle mass and density that occur with the liver transplant event, considering the extensive literature that notes sarcopenia is a risk factor for adverse outcomes. Potential transplant candidates present with a broad spectrum of nutritional and body composition conditions, often being deconditioned and malnourished. Body composition measurements to evaluate muscle mass as part of a nutritional assessment are often warranted [15, 16]. The ability to modify sarcopenia (and its outcomes) through nutritional or exercise intervention is a different subject [17]. Awareness of muscle mass/nutrition in the management of liver transplantation is essential to identify recipients at risk and for the design of therapeutic interventions [18, 19].

## 2 Methods

The institutional review board (IRB) at the University of Minnesota approved this study (STUDY00016254). A total of 710 patients underwent liver transplantation between 1/2016 and 8/2024. Of these 104 adult patients had CT scans within 90 days pre‐ and 90 days post‐transplant. PAI at L3 and mHU were measured using previously established methods [20]. For CT scans using IV contrast, a correction factor of −7.5 HU was used [13].

The CT muscle variables (PAI for muscle mass and mHU for muscle density/composition) of 104 recipients were stratified into quartiles for subsequent analysis. Within each quartile, the demographics, measured variable, with inclusion of the other variable were assessed before and after liver transplantation.

Descriptive statistics (means, standard deviations, medians, ranges for continuous variables; counts and percentages for categorical variables) were used to summarize the data overall and by groupings based on pre‐transplant PAI and mHU quartiles. Fisher's exact tests and one‐way analysis of variance (ANOVA) tests (or Kruskal–Wallis tests for length of stay) for categorical and continuous variables, respectively, were used to test associations between groups and demographic/characteristics. For adjusted analyses, multivariable linear regression models were used to test associations between demographic/characteristics and change in PAI or mHU. Paired *t*‐tests were used to test pre‐ to post‐transplant change in PAI and mHU. *p* values less than 0.05 were considered statistically significant. SAS V9.4 (SAS Institute Inc., Cary, NC) and GraphPad Prism v10.3.0 were used for analyses.

## 3 Results

The average age of the total cohort was 53 years (SD 10.9); of which 49% (*n* = 51) were male, and 76% (*n* = 79) were Caucasian (Table 1). The average laboratory MELD score was 30.4 (SD 7.8) at the time of transplant. Indications for liver transplant included: alcoholic cirrhosis (*n* = 45, 43.3%), metabolic dysfunction associated steatotic liver dysfunction (MASLD) (*n* = 17, 16.3%), hepatocellular carcinoma (*n* = 14, 13.5%), hepatitis B (*n* = 6, 5.8%), other (*n* = 10, 9.6%), unknown (*n* = 12, 11.5%). Median BMI was 29.8 (SD 7.4). 67.3% (*n* = 70) of patients were discharged home, 19.2% (*n* = 20) were discharged to a long‐term assisted care facility/skilled nursing facility (LTAC/SNF), 1.9% expired (*n* = 2), and 11.5% (*n* = 12) were discharged to an acute rehab facility. Median length of stay was 18 days (IQR 10.5–29). Patients who were discharged home had a median length of stay of 13 days, while those discharged to either an LTAC or acute rehab facility had a median length of stay of 28 days. The mean interval between pre‐ and post‐transplant CT scans was 45.8 ± 26.7 days for the total cohort and did not differ significantly between PAI quartiles (*p* = 0.99) or mHU quartiles (*p* = 0.34) (Table 1 and Table 2). Thus, differences in muscle mass and density changes across quartiles were not attributable to variations in scan timing.

**TABLE 1:** Summary of demographics/characteristics by PAI groups.

	Total *n* = 104	Pre‐PAI quartile 1 *n* = 26	Pre‐PAI quartile 2/3 *n* = 53	Pre‐PAI quartile 4 *n* = 25	*p* value^a^
Gender, *n* (%)					**0.0053**
F	53 (51.0)	19 (73.1)	27 (50.9)	7 (28.0)	
M	51 (49.0)	7 (26.9)	26 (49.1)	18 (72.0)	
Race, n (%)					0.1397
Native American	3 (2.9)	2 (7.7)	1 (1.9)	0	
Asian	12 (11.5)	4 (15.4)	4 (7.5)	4 (16.0)	
Black	10 (9.6)	5 (19.2)	4 (7.5)	1 (4.0)	
White	79 (76.0)	15 (57.7)	44 (83.0)	20 (80.0)	
Age, mean (SD)	53.2 (10.9)	52.7 (12.1)	51.2 (10.1)	58.2 (9.9)	**0.0274**
BMI, mean (SD)	29.8 (7.4)	30.1 (9.1)	28.7 (6.3)	31.7 (7.7)	0.2413
MELD, mean (SD)	30.4 (7.8)	30.4 (8.6)	31.4 (7.1)	28.4 (8.4)	0.2768
Indication for LT, *n* (%)					0.2989
Alcohol cirrhosis	45 (43.3)	9 (34.6)	27 (50.9)	9 (36.0)	
HCC	14 (13.5)	5 (19.2)	4 (7.5)	5 (20.0)	
Hepatitis B	6 (5.8)	2 (7.7)	4 (7.5)	0	
MASLD	17 (16.3)	3 (11.5)	8 (15.1)	6 (24.0)	
Other	10 (9.6)	3 (11.5)	3 (5.7)	4 (16.0)	
Unknown	12 (11.5)	4 (15.4)	7 (13.2)	1 (4.0)	
Hosp LOS, median (IQR)	18 (10.5–29)	18.5 (12–29)	19 (10–30)	15 (10–22)	0.2471
Disposition, *n* (%)					0.8339
Acute rehab	12 (11.5)	3 (11.5)	7 (13.2)	2 (8.0)	
Expired	2 (1.9)	0	2 (3.8)	0	
Home	70 (67.3)	16 (61.5)	36 (67.9)	18 (72.0)	
LTAC	20 (19.2)	7 (26.9)	8 (15.1)	5 (20.0)	
Pre‐mHU, mean (SD)	35.47 (8.78)	35.25 (8.85)	36.03 (8.73)	34.50 (9.11)	0.7693
Post‐mHU, mean (SD)	33.00 (9.78)	32.28 (11.53)	33.11 (9.12)	33.50 (9.52)	0.9001
mHU change, mean (SD)	−2.47 (9.17)	−2.98 (9.57)	−2.92 (9.66)	−1.00 (7.77)	0.6602
Pre‐PAI, mean (SD)	7.94 (2.64)	5.07 (0.82)	7.62 (0.86)	11.58 (2.16)	**<.0001**
Post‐PAI, mean (SD)	6.99 (2.44)	4.75 (1.14)	6.72 (1.64)	9.87 (2.04)	**<.0001**
PAI change, mean (SD)	−0.95 (1.48)^b^	−0.32 (1.02)	−0.90 (1.38)	−1.71 (1.77)	**0.0028**
Days between CT, mean (SD)	45.8 (26.7)	46.2 (26.5)	45.8 (25.9)	45.2 (29.4)	**0.9923**

^a^ *p* values are from Fisher's exact tests for categorical variables, ANOVA tests for continuous variables and a Kruskal–Wallis test for length of stay. *p* < 0.05 considered statistically significant.

^b^ Paired *t*‐test *p* value < .0001.

**TABLE 2:** Summary of demographics/characteristics by mHU groups.

	Total *n* = 104	Pre‐mHU quartile 1 *n* = 26	Pre‐mHU quartile 2/3 *n* = 52	Pre‐mHU quartile 4 *n* = 26	*p* value^a^
Gender, *n* (%)					0.5612
F	53 (51.0)	11 (42.3)	27 (51.9)	15 (57.7)	
M	51 (49.0)	15 (57.7)	25 (48.1)	11 (42.3)	
Race, *n* (%)					0.5489
Native American	3 (2.9)	2 (7.7)	1 (1.9)	0	
Asian	12 (11.5)	3 (11.5)	8 (15.4)	1 (3.8)	
Black	10 (9.6)	2 (7.7)	5 (9.6)	3 (11.5)	
White	79 (76.0)	19 (73.1)	38 (73.1)	22 (84.6)	
Age, mean (SD)	53.2 (10.9)	55.2 (9.7)	53.5 (11.2)	50.9 (11.2)	0.3496
BMI, mean (SD)	29.8 (7.4)	31.0 (8.1)	29.7 (7.5)	28.6 (6.6)	0.5225
MELD, mean (SD)	30.4 (7.8)	31.1 (8.4)	30.9 (6.7)	28.8 (9.3)	0.4617
Indication for LT, *n* (%)					0.8093
Alcohol cirrhosis	45 (43.3)	16 (61.5)	19 (36.5)	10 (38.5)	
HCC	14 (13.5)	2 (7.7)	8 (15.4)	4 (15.4)	
Hepatitis B	6 (5.8)	1 (3.8)	4 (7.7)	1 (3.8)	
MASLD	17 (16.3)	4 (15.4)	8 (15.4)	5 (19.2)	
Other	10 (9.6)	1 (3.8)	7 (13.5)	2 (7.7)	
Unknown	12 (11.5)	2 (7.7)	6 (11.5)	4 (15.4)	
Hosp LOS, median (IQR)	18 (10.5–29)	27 (12–34)	19 (10–28)	13 (9–24)	0.0799
Disposition, *n* (%)					0.9018
Acute rehab	12 (11.5)	3 (11.5)	7 (13.5)	2 (7.7)	
Expired	2 (1.9)	0	2 (3.8)	0	
Home	70 (67.3)	17 (65.4)	33 (63.5)	20 (76.9)	
LTAC	20 (19.2)	6 (23.1)	10 (19.2)	4 (15.4)	
Pre‐PAI, mean (SD)	7.94 (2.64)	8.21 (2.18)	7.59 (3.01)	8.35 (2.22)	0.4072
Post‐PAI, mean (SD)	6.99 (2.44)	7.44 (2.40)	6.50 (2.54)	7.50 (2.15)	0.1292
PAI change, mean (SD)	−0.95 (1.48)	−0.77 (1.60)	−1.09 (1.51)	−0.85 (1.31)	0.6262
Pre‐mHU, mean (SD)	35.47 (8.78)	23.31 (4.83)	36.36 (3.31)	45.84 (2.22)	**<.0001**
Post‐mHU, mean (SD)	33.00 (9.78)	26.09 (9.02)	33.53 (9.51)	38.83 (6.52)	**<.0001**
mHU change, mean (SD)	−2.47 (9.17)^b^	2.78 (8.44)	−2.83 (9.39)	−7.01 (6.72)	**0.0004**
Days between CT, mean (SD)	45.8 (26.7)	46.4 (27.3)	42.4 (26.5)	51.8 (26.2)	**0.3387**

^a^ *p* values are from Fisher's exact tests for categorical variables and ANOVA tests for continuous variables. *p* < 0.05 considered statistically significant.

^b^ Paired *t*‐test *p* value = 0.0071.

### 3.1 Loss of PAI Was Greatest in Patients Who Had the Greatest Values Preoperatively

**FIGURE 1:**
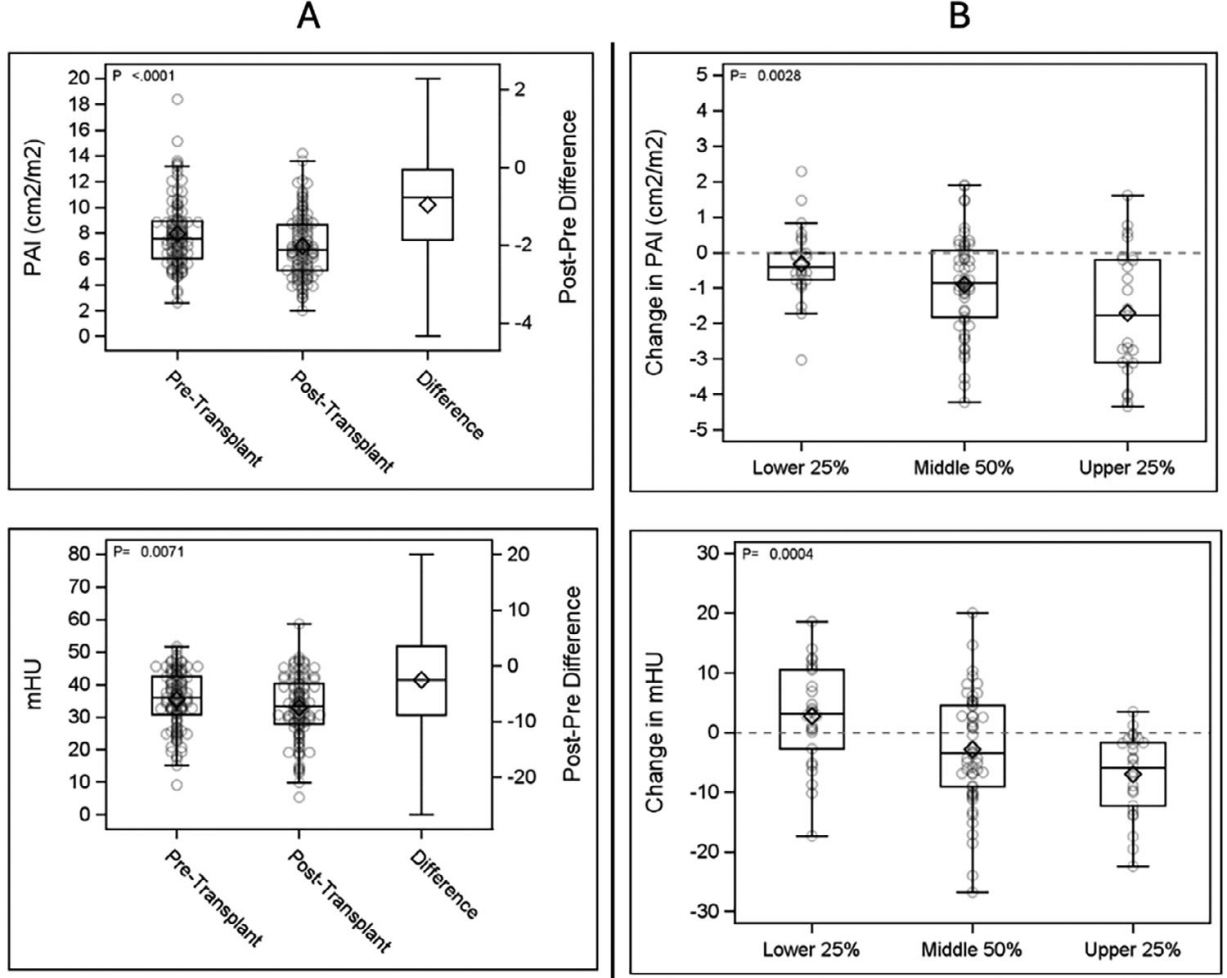
(A) Box and whisker plot of pre‐ and post‐transplant PAI and mHU. (B) Box and whisker plot of the change in PAI (upper figure) and mHU (lower figure) based on quartiles being the upper 25%, the middle 50%, and the lower 25%.

The mean PAI pre‐transplant for the cohort was 7.94 cm^2^/m^2^ (SD 2.64), and post‐transplant was 6.99 cm^2^/m^2^ (SD 2.44), representing a decrease of 0.95 cm^2^/m^2^ (12%; *p* value < 0.0001) (Table 1; Figure 1). A striking difference was noted when the aggregate PAI was divided into quartiles. The mean upper quartile PAI was 11.58 (SD 2.16), the middle two quartiles mean PAI was 7.62 (SD 0.86), and the lowest quartile mean was 5.07 (SD 0.82) cm^2^/m^2^. In the upper quartile, the majority were male (72%) and trended older age (mean 58.2 years, SD 9.9) vs the lower quartile, a female majority (73%) and somewhat younger age (mean 52.7 years, SD 12.1). The MELD scores were clinically similar between upper and lower quartiles (*p* = NS). Changes in post‐transplant PAI significantly differed depending on the pre‐transplant PAI quartile. The upper PAI quartile had an average PAI reduction of 15% (11.58 to 9.87 cm^2^/m^2^ (−1.71cm^2^/m^2^, *p* < 0.0001). The mid two PAI quartiles were reduced −12% from pre‐ to post‐transplant, 7.62 to 6.72 (−0.9) cm^2^/m^2^ (*p* < 0.0001). However, recipients within the lowest PAI had a more modest reduction in muscle mass, but still statistically significant, −6% (5.07 to 4.75 cm^2^/m^2^, *p* < 0.003) (Table 1).

### 3.2 Loss of mHU Was Greatest in Patients Who Had the Greatest Values Preoperatively

Segregating the cohort by muscle density (mHU), no demographic elements were statistically associated with muscle density. The average mHU pre‐transplant for the cohort was 35.47 (SD 8.78), and post‐transplant was 33.00 (SD 9.78), representing a mean decrease of 2.47 (7%; *p* value = 0.0071) (Table 2, Figure 1). However, as with PAI, there was variation by quartile. The mean mHU was 23.31 in the lowest quartile, 36.36 in the middle quartiles, and 45.84 in the upper quartile. Pre‐transplant muscle density/mHU had little correlation with PAI, as noted in Table 2 (pre‐transplant *p* = 0.41). Post‐transplant mHU change differed between quartiles. The upper quartile had −15% mHU change, pre‐45.84 and post‐38.83 mHU (−7.01 mHU), the middle two quartiles a lesser change, −2.83 mHU (−8%), and the lowest mHU quartile had an *increase* in mean mHU, 23.31 to 26.09 ( + 12%).

### 3.3 Clinical Variables Such as Sex and Age Did Not Modify PAI or mHU Loss. Only Pre‐Transplant PAI and mHU Were Predictive of Post‐Transplant Changes

Males typically have more muscle than females, which is reflected in the percentage of males within the highest PAI quartile. However, when analyzed according to sex, there was no statistically significant difference in PAI percentage lost from pre‐ to post‐transplant. On average, males lost 9.73% of their pre‐transplant PAI, whereas females lost 12.09% (*p* value = 0.8584). Furthermore, there was no significant difference in mHU percentage lost across transplant when stratified by sex. Males lost an average of 4.57% of mHU and females lost an average of 3.33% mHU (*p* value = 0.9230). There were no statistically significant differences in gender, race, age, BMI, MELD, LOS, indication for LT, or disposition on multivariable linear regression analysis (Table 3). There were no statistically significant changes between pre and post LT mHU when quartiles were divided on pre‐LT PAI, and no statistically significant changes in pre or post PAI when the cohort was stratified by pre‐LT mHU. Applying multivariable linear regression to the clinical variables demonstrated that the only variables associated with post‐transplant changes in either psoas/core muscle volume (PAI) or muscle density (mHU) were the pre‐transplant PAI and mHU, respectively (Table 3).

**TABLE 3:** Associations of demographics/characteristics with change in PAI and mHU—Results of multivariable linear regression models.

	Outcome: PAI change		Outcome: mHU change	
Covariates	Estimate (SE)	*p* value	Estimate (SE)	*p* value
Female gender	−0.28 (0.32)	0.3809	1.45 (1.88)	0.4419
Race (ref = White)		0.6169		0.5367
Native American	−0.75 (0.83)	0.3687	7.05 (5.22)	0.1803
Asian	−0.44 (0.50)	0.3802	−0.69 (3.09)	0.8226
Black	0.08 (0.62)	0.9030	−1.75 (3.87)	0.6525
Age	−0.02 (0.02)	0.2651	0.07 (0.10)	0.4648
BMI	−0.01 (0.02)	0.5342	−0.20 (0.14)	0.1616
MELD score	−0.03 (0.02)	0.0943	0.25 (0.13)	0.0506
LOS	−0.001 (0.01)	0.8825	−0.08 (0.06)	0.1495
Indication for LT (ref = AC)		0.2895		0.3208
HCC	0.17 (0.51)	0.7489	6.66 (3.21)	0.0406
Hepatitis B	0.28 (0.82)	0.7309	6.82 (5.15)	0.1892
MASLD	0.10 (0.45)	0.8144	0.81 (2.77)	0.7695
Other	−1.12 (0.53)	0.0372	2.96 (3.26)	0.3664
Unknown	−0.37 (0.49)	0.4443	3.77 (3.04)	0.2182
Disposition (ref = Home)		0.7961		0.1764
Acute rehab	−0.26 (0.52)	0.6245	−4.48 (3.23)	0.1698
Expired	−0.80 (1.07)	0.4548	−11.67 (6.62)	0.0816
LTAC	−0.31 (0.41)	0.4537	−3.62 (2.53)	0.1561
Pre‐measure	−0.26 (0.06)	**<.0001**	−0.49 (0.10)	**<.0001**

*Note:* Results are from multivariable linear regression models. Pre‐measure is the pre‐transplant PAI or mHU value.

## 4 Discussion

Not surprisingly, this analysis demonstrated that the cohort's core body muscle mass (measured by PAI) and muscle density (measured by mHU) were diminished following liver transplantation. What was not expected was the amount of variation noted when the measures were segregated into pre‐operative quartiles. Recipients with the greatest muscle mass pre‐transplant had the highest percentage of muscle reduction (15%) post‐transplantation. Those with the lowest pre‐transplant PAI had the least reduction post‐transplant (6%). Muscle density (HU) did not correlate with PAI or other demographic variables but demonstrated significant post‐transplant changes. In the highest quartile pre‐transplant, mHU was reduced by 15%, the mid‐quartiles HU reduced by 8%, but the lowest pre‐transplant quartile had an increase in muscle density of  + 12%. Whether this diversity in muscle changes between the pre‐ and post‐transplant state is clinically relevant was not the intent of this analysis. However, the recipient's pre‐transplant clinical condition and the timing of the CT assessments impact the assessment of core body muscle. Importantly, the interval between pre‐ and post‐transplant CT imaging did not differ across quartiles, suggesting that the observed differences in PAI and mHU changes were not confounded by variation in scan timing. This supports that the magnitude of muscle change reflects intrinsic physiological differences rather than differences in follow‐up duration.

Liver transplantation is reserved for individuals with few other options. Skeletal muscle mass serves as a nitrogen reservoir to support the metabolic demands of recovery. Liver transplantation induces a significant catabolic and anabolic demand that, when adequately met, permits the recipient to recover in time. [21, 22]. This analysis highlights the statistically significant variations in the change trajectory of the recipient muscle when stratified by either PAI or mHU. Recipients with abundant muscle mass and density experience the greatest reduction in volume and density after liver transplantation. However, this is the subpopulation identified in the literature as optimal candidates. LT candidates with the least PAI and lowest mHU have a proportionally lesser muscle mass loss and an increase in muscle density. We presume that the nitrogen demand is similar during the peri‐transplant period, irrespective of the quantity and quality of the recipient muscle. Whether mobilization of other protein stores within the body occurs is unknown, but it appears that in the lowest PAI quartile, relatively little skeletal muscle loss occurs.

The decision to utilize PAI rather than skeletal muscle index (SMI) derived from machine learning algorithms of total abdominal muscle area was intentional. While total SMI obtained from full abdominal segmentation is a widely used metric, PAI was selected for its clinical practicality, reproducibility, and strong correlation with total skeletal muscle mass on CT. Prior comparative analyses [14] have demonstrated that PAI and total SMI provide similar stratification for liver transplant candidates and serve as a reliable surrogate for total SMI in this population. Importantly, the relative ease and speed of calculating PAI make it a clinically useful measure that can be readily incorporated into routine imaging review. While possibly more available in the future, currently, not all centers have access to automated deep learning algorithms or specialized body composition analysis software necessary to perform total SMI assessment. In summary, we feel that the practicality, reproducibility, and broader applicability of PAI in both research and clinical settings make it a more ideal measurement for this study. Nevertheless, the use of PAI rather than total SMI may limit direct comparison with some prior studies, and results should be interpreted within this methodological context.

While this study demonstrates the statistical difference within the cohort of muscle changes when stratified by either PAI or mHU, the small number and diversity of recipients and diseases did not lend themselves to a more in‐depth analysis. There are, of course, other limitations; while all recipients received the “routine” standard of care, including protein supplementation, occasional enteric feeding, and physical therapy, the extent to which nutritional and physical therapy goals were met was unavailable. Whether the muscle changes could be abrogated by nutritional or therapy interventions could not be assessed. While frailty and sarcopenia are often linked, this study could not associate physical assessment of pre‐transplant frailty with sarcopenia. There were too few frailty measures within the pre‐transplant time window. In addition, no direct measures of muscle function or strength (e.g., grip strength, gait speed, or validated frailty indices) were available for this cohort. The absence of functional assessment limits interpretation of whether the observed changes in muscle mass and density translated into clinically meaningful impairment, although it may be safely assumed that functional scores would have decreased in the immediate post‐transplant period. Consequently, while PAI and mHU provide objective measures of structural change, they may not fully capture the physiologic or functional recovery of the post‐transplant patient. Our study demonstrates significant alterations in post‐transplant muscle mass and density that have the likelihood of altering the rate of functional recovery. Incorporating standardized measures of muscle function in future studies will be essential to link imaging‐based sarcopenia metrics with patient‐centered outcomes and to refine their translational relevance. Conceptually, frailty and sarcopenia should have a strong correlation; however, it was not possible to assess this correlation with this cohort. It should be remembered that this was a retrospective, observational study that demonstrated significant findings, but was without causal hypotheses or the impact of therapeutic interventions.

There is no agreed‐upon value for PAI correlating to sarcopenia; however, it is reasonable to state that the lower the PAI, the closer to sarcopenia an individual becomes [23]. This analysis did not assert an absolute degree of PAI as sarcopenic; rather, the cohort quartiles were compared. A further step that will be critical in the future of the study of sarcopenia would involve establishing an agreed‐upon value of PAI for sarcopenia that correlates to adverse outcomes.

## 5 Conclusion

The data obtained from this study suggest that recipients with more skeletal muscle mass undergoing LT lose more muscle postoperatively compared to those who begin with less core muscle mass (psoas). Furthermore, it appears that the density of the skeletal muscle decreases similarly depending on the starting quartile, as evidenced by a decrease in average mHU. Published literature notes that post‐LT adverse outcomes/mortality risk is highest for those recipients within the lowest muscle quartiles, suggesting that muscle change/loss is not a major determinant of adverse outcomes. It remains to be discerned whether factors associated with pre‐transplant sarcopenia can be significantly modified by post‐LT nutritional or physical therapy interventions. Our findings suggest that the endogenous protein metabolism may be modulated by the quantity and quality of core muscle at the time of LT. Conventional wisdom would suggest that early integration of physical therapy, occupational therapy, and dietary consultations is the best available tool to decrease the extent of sarcopenia experienced post liver transplant [24, 25].

## Author Contributions

Concept and design, data analysis and interpretation, drafting, critical revision of article, approval of article: Michael G. Megaly. Concept and design, data collection, data analysis and interpretation, drafting, approval of article: William C. Miller. Concept and design, data collection, data analysis and interpretation, drafting, revision, approval of article: Jessica Thul. Data analysis and interpretation, approval of article: Peter Gullickson. Data analysis and interpretation, drafting, approval of article: Abraham J. Matar. Drafting, revision, critical revision of article, approval of article: Matthew Wright, David Mathews, Jessica Fisher, Heidi Sarumi, Michael Dryden. Statistical analysis, data analysis and interpretation, critical revision of article, revision, approval of article: Levi Teigen. Statistical analysis, data analysis and interpretation, critical revision of article, approval of article: Scott Lunos. Concept and design, data analysis and interpretation, critical revision of article, revision, approval of article: Timothy L. Pruett.

## Disclosure

Research reported in this publication was supported by the National Center for Advancing Translational Sciences of the National Institutes of Health, Award Number UM1TR004405. The content is solely the responsibility of the authors and does not necessarily represent the official views of the National Institutes of Health.

## Conflicts of Interest

The authors declare no conflicts of interest.

## Data Availability

The data that support the findings of this study are available from the corresponding author upon reasonable request.
